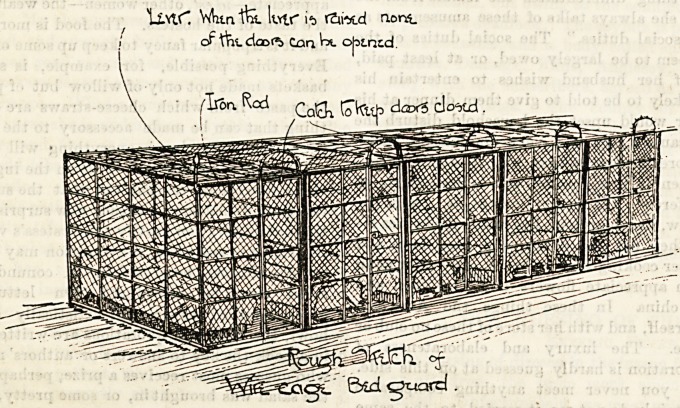# The Hospital Nursing Supplement

**Published:** 1894-12-01

**Authors:** 


"The Hospital" Nursing Mirror.

Being the Extra Nursing Supplement of "The Hospital" Newspaper.

[Contributions for this Supplement should be addressed to the Editor, The Hospital, 428, Strand, London, W.C., and should have the word "Nursing" plainly written in left-hand top corner of the envelope.]

### News from the Nursing World.

### FEASTS AND FESTIVALS.

Notices of Christmas entertainments in hospitals and other institutions will be inserted this year as usual in the columns of The Hospital, but it is essential that they be received as soon as possible after the events have taken place. It is also desirable that announcements of approaching festivities intended for publication should be sent in at an early date. Some of our correspondents seem to fancy that newspapers are printed on the day of publication.

### CHARING CROSS HOSPITAL.

"But where is this hospital?" inquired one to whom post office, station, and many other local landmarks had long been familiar. Really it seems as if useful little Charing Cross Hospital were chronically forgotten by the thousands who daily pass by it. The convenience of the site it occupies is quickly acknowledged in those emergencies which fill the wards. Patients do not wish to "overlook" the place when they have once experienced the care and skill unstintedly granted to them there. A new visitor passing in always feels surprised at the brightness of the rooms and the spotless floors; the cheerful general appearance of the building, whose unobtrusive exterior has probably failed to arouse his interest. Certainly Charing Cross Hospital ought not to be forgotten, and any one wishing to add to its funds or to give gifts in kind cannot do better than go and judge for himself of its condition. The majority of patients are men, and it is obvious that clothes are in urgent demand to fit out the poor fellows when they are convalescent after accidents which, besides physically injuring the individual, generally utterly ruin his clothes. If a breadwinner is long out of work, it is well-nigh impossible for his wife to supply him with all that is necessary.

### AT WINDSOR.

Not only donations but much personal interest have been bestowed by Her Majesty on the sufferers from the recent floods. When the gas supply failed, lamps were supplied by her, as well as quantities of soup, piles of beautiful blankets, shawls, and other things. The recipients, in all their discomforts, were cheered by knowing that the Queen was thinking of them, and planning early and late for the relief of the widespread distress. The details of each day's doings have been doubtless read with deep interest by many nurses, and one of the most pathetic incidents, to our mind, is that of the delicate woman, with curvature of the spine, who retreated to the top room of a house where she remained alone for many hours without food, fire, or drinking water. Many methods of succour were attempted, but all failed until a canoe was obtained, and cleverly steered through a narrow opening and floated within reach of the poor creature, to whom it brought a supply of the necessaries of which she was in such urgent need.

### FANCY VERSUS PLAIN WORK.

The promotion of occupations which interest as well as employ the poor in workhouse infirmaries is a movement to appeal to the sympathy as well as to the common sense of the public. Days and weeks passed without anything to do naturally have a depressing effect on those who have led active lives. Yet it is surely unwise for fancy work to take the place of plain sewing in female wards, although it is well for the men to be supplied with such materials as their inexperienced fingers can manipulate, and it is pleasant to see the really artistic work carried out by male patients under the Brabazon scheme. We must, however, protest against women patients being encouraged to make fancy articles when they might more profitably be engaged in making nice little frocks and pinafores for wee girl paupers or blouses for the small boys. An infirmary matron with taste and skill can impart attractions to all such things as these. It is not fair to ratepayers that "ready-made garments" should be bought for the inmates whilst the latter are kept busy "doing wool-work." They might be quickly and easily instructed in sewing flannel vests or calico nightgowns!

### OUR CHRISTMAS COMPETITION.

"I am getting quite anxious about Christmas presents for the patients, none have come in yet," sighed a matron. "I thought you would be over-burdened with such things," said the surprised visitor, "at the Children's Hospital near my house, they say they don't know what to do with half that is sent them!" Because one or two miniature institutions have over much it seems hard that the majority of large ones are left to depend for Christmas gifts on the precarious charity of chance-comers! Sisters and nurses as well as matrons have to supplement this inadequate supply by the work of their own hands, and by contributions from slender purses. Such things ought not to be. The presents should surely come from those who cannot give personal service. "I never know what to give" is no excuse when everything is welcome. A useful garment is needed for each woman, and for the men flannel shirts and vests are specially in demand. Stockings and socks, shoes and boots are needed. "We never get nearly enough things for the men," says another matron, "and I would not have them left out on any account. Last year I did not know how to manage, so I made up parcels for all the married ones, containing little garments for their children. Their pleasure in having these to hand over to their wives on Christmas afternoon was great." Our readers are busy preparing Christmas gifts for friends and relations. Won't they extend their preparations by adding just a few more articles to the parcels which should reach this office by December 18th? All cannot gain prizes, but nevertheless each offering will gladden the heart of some sick brother or sister, to whom, perchance, the receipt of a Christmas-box is a rare luxury. The prizes offered are: 20s. for the most serviceable dressing gown; 10s. for the best flannel shirt; 7s. 6d. for the best flannel petticoat; 7s. 6d. for the best over petticoat; 7s. 6d. for the best bed jacket; 5s. for the best knitted pair of men's socks; and 2s. 6d. for the second best pair. Those who do not care to compete are invited to contribute garments of all descriptions to Nursing, care of Editor, 428, Strand, by December 18th. Competitors should put the words "Needlework Competition" outside, and their full names and addresses inside parcels.

### NURSING SISTERS IN THE AMRY.

Nursing sisters are entitled to thirty days' leave of absence every year, and they have to retire as soon as they reach the age of sixty, when they are pensioned at a rate to accord with the duration of their service. The pay of ladies filling the responsible posts of superintendents commences at £150 and rises to a maximum of £200. The nursing sisters have £30 to begin with, which is gradually increased to £50. Uniform and other allowances are made. It is now required that candidates must have had three years' training in a general hospital, that they shall be over twenty-five and under thirty-five years of age, and have certificates of health and efficiency and a recommendation from the Matron under whom they have worked.

### NURSES IN EAST LONDON.

The East London Nursing Society wants more financial support to enable it to carry on and extend its sphere of usefulness. The district nurses work in Hoxton, Poplar, Limehouse, Shadwell, Ratcliffe, Wapping, and Stepney. During the last year 87,382 visits have been paid to 4,259 sick persons. Subscriptions will be gratefully received and acknowledged by Mr. A. W. Lacy, 49, Philpot Street, Commercial Road, E.

### NURSES AT SWANSEA.

The nursing staff at Swansea General Hospital numbers a matron, five sisters, and seventeen nurses, to eighty-three patients, for that seems to be the average of beds occupied. We congratulate the hospital upon obtaining so adequate a staff, which, however, we believe has only been rendered possible by the introduction of paying probationers. The Hospital committee and the matron are to be congratulated on the example they are able to set in this department.

### PROGRESS IN LIMERICK.

The Governors of Limerick County Infirmary have begun the reorganisation of their nursing department by installing a trained nurse. Miss J. F. Mayne (Sister Frances) was the first head nurse employed at King's County Infirmary under the trained matron appointed there two years ago, and her work has been so satisfactory that Limerick County Infirmary is fortunate in securing her services. It is probable that the latter institution intends to follow the excellent example set by King's County, and that more posts will presently be offered to trained and experienced helpers.

### QUEEN'S NURSE AT INVERNESS.

The Inverness branch of the Queen's Jubilee Institute held its third annual meeting on the 16th ult., which was well attended. Provost Ross presided, and appealed for fresh subscribers, as the amount at present received only covered expenses, leaving no balance for emergencies. Dr. Norman Macleod made an interesting speech, in the course of which he said: "No doubt human nature, especially stingy human nature, was very fertile and ingenious in discovering reasons for not supporting any scheme for the physical, moral, or spiritual amelioration of the condition of their fellow creatures, but he could say that he never knew any person who had ventured to suggest that a nurse for the sick poor was a superfluity." The efficient work of Nurse Nicolson was warmly acknowledged. Dr. Black, complimenting her on the remarkable improvements noticed in the condition of sick rooms she had visited. The necessity for engaging the services of an additional nurse as soon as the funds permitted was urged upon those present at the meeting.

### AMONGST THE FISHERMEN.

The fishing is over on the Labrador, and most of the people have returned to Newfoundland after an unprofitable season. The hospital at Indian Harbour is closed for the winter, having received twenty in-patients in three months and five hundred and eighty out-patients. It gives some idea of the surroundings to which these people are accustomed when we hear of their looking upon the bare, homely little hospital as "so beautiful, why sure this must be quite like a palace!" So poor are the people that many infants die, and few, indeed, have adequate food or clothing. Through the Mission to Deep Sea Fishers medical skill is now placed within their reach during the summer season, and gifts of clothing have been forwarded. The workers have returned to England until spring, when they will pass the ice once more. They cannot fail to realise that they have saved the lives of many breadwinners who would probably have died without a doctor's skill and nurse's care. The little hospital at Battle Harbour has received twenty-seven in-patients and four hundred and sixty out-patients, and has been much appreciated.

### SHORT ITEMS.

An appeal is made for subscription s and also for invalid appliances by the promoters of the Wallsend Nursing Association, who hope to install a trained district nurse for the sick poor; and appliances to form a loan collection for their use.—Half the proceeds of the Barry Poultry Show have been promised to the Barry Nursing Institute.—The committee of the Bridport General Nursing Society reports that Nurse Bradley has made 2,769 visits during the year and that her work has given entire satisfaction.—A lecture entitled "Hints on the Management of Children's Health and Physical Development" was recently given in connection with the Parents' National Educational Union at Hastings by Miss Amy Fiennes Twisleton, nursing sister at the Bexhill Metropolitan Convalescent Home.—The Management Committee of the Joint Hospital, Calverley, have decided that when temporary extra help is required the Matron shall engage nurses from the Fulneck Nurses' Institution.—Accommodation for the private nurses as well as for the resident nursing staff is provided by the new nurses' home at Cumberland Infirmary.—Last week's *Nursing Talk* at the Trained Nurses' Club was given by Mrs. Langton Hewer, the subject of "Diet and Medicines for Children" being most ably treated by the authoress of the well-known volume "Our Baby."—There are twenty-eight nurses working amongst the sick in Newport, Isle of Wight; beef tea, brandy, and milk are supplied, under the doctors' directions, through the relief fund, and further subscriptions are urgently needed in the present sad epidemic.—A Samaritan fund in connection with the Longton Sick Nursing Association has been inaugurated by a gift of £100 from Sir Smith Child, Bart.

### Antiseptics.

By F. R. HUMPHREYS, L.R.C.P. Lond., M.R.C.S.E.

In the application of antiseptics we wage war against the lowest form of vegetable organism, and, as in other wars, it is he who pays most attention to detail who wins the fight. Our bodies are at constant war with the hosts of unseen germs which surround us, constantly endeavouring to break through our fortifications, and now and then succeeding, and they find their way into the tissues, blood, &c., and have to be attacked there under still greater difficulties.

We may divide the science of antiseptics under two main heads: 1. The attack on the germs. 2. The antidotal treatment of their poisonous excretions.

As to the germs, these have to be attacked both when external to the body and merely threatening it, and also when they have obtained entrance to it or when they are in the very act of so doing, as when on the surface of an open wound. The attack on the germs altogether outside the body, and merely a standing menace to it, belongs strictly to the science of sanitation.

The germs are attacked on the general rules which a minute investigation into their ways and habits has afforded, and there should be no more an element of chance in our preventive treatment than there should be with a chemist in his laboratory, of an experiment failing through the boiling over of a test tube. Unfortunately we have not yet reached this state of perfection, and the germs cause a lot of disease as a consequence. Germs on the surface of a wound are attacked more in detail than those in a sewer can be, and the study of such matters is now leading us to expect that as a matter of course there shall be no germs on the surface of the wound when it is finally closed up by dressings. We are more and more learning that suppuration, inflammation, and free discharges from a wound are the sure indications of some failure in the antiseptic treatment. The principal trouble in all antiseptic treatment whether of sewers or of wounds, lies in the destruction of the spores or eggs of the germs. These little bodies are covered externally by a very resisting envelope or shell, and the great difficulty lies in penetrating this covering so as to effect the death of the germ within. Common soap and water appear to have a very good effect in this direction, the envelope dissolving in the excess of alkali present in common soaps. Heat, if prolonged and high, will also destroy them, and full exposure to air and moisture makes them decay. Ordinary antiseptic solutions have only this effect, that they prevent their development, hence it is that sterilised dressings which have no antiseptic in them appear to be of less value, in English hands at any rate, than dressings containing an antiseptic. Still such dressings are very largely used abroad, and can be readily prepared anywhere. For example, linen, which has been thoroughly washed and boiled, is put into a tin box—for instance, a biscuit-tin—and this is wrapped in paper and placed in an oven in which the temperature is sufficiently high to slightly scorch the paper. It is kept there for about half an hour, and the dressings are used at once on opening it. Each time they are required they should be rebaked. The centre of a piece of medicated cotton wool is used to cover the dressings, and the whole closely bound on, or any cotton-wool may be used after it has been well baked as directed. Antiseptic solutions have a stimulating effect on the wound, which is not altogether desirable, as it tends to cause a free flow of fluids, and these will become infected if they escape from the dressings, and the septic infection will spread under the edges of the dressings to the wound. Sterilised water is therefore much used instead. In order to prevent much discharge taking place, all deep wounds are sewn up from the bottom, and not merely at the surface, and then no drainage tube need be inserted. The wound, which, of course, must be thoroughly aseptic to begin with, is not disturbed so long as the temperature keeps normal, and the dressings do not slip.

It will be readily understood that septic organisms abound in hospitals aud places in which wounds are habitually treated, and that there is a very great danger of the surface of a wound becoming infected by the germs which are being wafted about in the air; but in private houses there is not the same danger, and whereas in some hospitals it might be absolutely necessary, even in the case of operative wounds where the skin, &c., had been thoroughly prepared beforehand, to use antiseptic as opposed to aseptic dressings, yet in private practice the latter might do equally well.

Nowadays we are not satisfied with attacking the germs of acute diseases prior to their having obtained ingress to the body, but we attack them after they have stormed the breach. In the process of getting into the tissues they are met by the phagocytes, the protecting army of the human body. From the tissues, at the point where the germs are attacking, are set free multitudes of cells which boldly attack the germs, and to their aid come cells from all parts of the body, so that the tissues which form the battlefield become distended with the cells and the serum which nourishes them and allows them to move about. The result of this is inflammation of the part, and thus inflammation is a beneficial and not a purely destructive process. We assist these phagocytes by the administration of antiseptics by the mouth, or by injection of them at the spot where the inflammation is taking place. Thus in typhoid fever we give iodoform, naphthaline, carbolic acid, and similar drugs by the mouth, in the hope that they may reach the inflamed glands in the intestines, and there assist the defending armies. Quinine is also a powerful antiseptic, and everyone knows how useful it is in fevers. The treatment of germs within the body is, however, still in its infancy, and we have much to learn about it.

### Where to Go.

PORTMAN ROOMS.—Subscription dance in aid of the East London Hospital for Children, Shadwell, on Tuesday, December 18th.

SALE of Work at Trained Nurses' Club on December 6th and 7th. Contributions for stalls should be sent in as soon as possible, and articles for the provision stall will be gratefully received. Many novel entertainments have been organised in connection with the sale, and a successful meeting is anticipated.

TRAINED NURSES' CLUB, 12, BUCKINGHAM STREET.—Nursing Talk. Subject, "Infectious Diseases of Children," by Mrs. Rowland Humphreys, at three p.m., Tuesday, December 4th. Fee for single lecture, 1s. 6d.

HOSPITAL FOR SICK AND INCURABLE CHILDREN, CHEYNE WALK, CHELSEA.—Tuesday and Wednesday, December 4th and 5th, from two to five p.m. Exhibition of work done by the children during the year. The work is not for sale.

ANNUAL ball in aid of the funds of the Royal Free Hospital, Gray's Inn Road, will take place on Wednesday, December 12th, at Holborn Town Hall.

IDEAL CLUB, 185, TOTTENHAM COURT ROAD.—On Thursday, December 6th, at eight p.m., Mr. H. Roberts will speak on "Public Control of Hospitals."

### Presentations.

A HANDSOME chair and oak table were recently presented to Mr. Geoffry Cross by the house surgeons, secretary, and nursing staff of the Sheffield General Infirmary on his resignation of the post of assistant house surgeon in that institution.

MISS E. L. CHURCHILL, on resigning the post of Parish Nurse of Timperley, Cheshire, was presented with a set of dessert knives and forks in case, a set of afternoon tea-spoons, and a revolving silver breakfast dish, with the following inscription: "To Nurse Churchill, on her leaving the parish of Timperley, a farewell offering from friends. In recognition of her invaluable work amongst the sick and poor."

### Asylum News and Nursing.

[Contributions to this section should be addressed to The Editor, 428, Strand, W.C., and have the words "Asylum News" written in left-hand bottom corner of the envelope.]

### AN ASYLUM FROM WITHIN.—II.

My first day's work left a vivid impression upon me, and yet when I look back I cannot say that there were any very unusual incidents in it. It was strange to hear criticisms free and very candid passed upon my appearance and dress. One said, "She looks a decent girl, but she'll soon be made as bad as the rest." Another, "The idea of putting a girl like that over us women." My charge nurse was slightly under middle height, but broad and strongly built. Though not good-looking, she seemed good-natured and capable. It needed an able woman to have several nurses under her, and so many patients in her charge. What a nurse will be, depends more upon the charge nurse she is under than anything else. She gave me to understand, that at first my duties would consist of errands, and odd jobs, until I became acquainted with the place and the people. She strongly advised me to lose no time in learning the patients' names, and little peculiarities, adding significantly, "for your own sake." It was not long before I found out what she meant. She then sent me to see the matron in her room, who gave me a copy of the rules of the institution, and a great deal of good advice, to which I am afraid I paid little attention. On my return to the ward, the charge nurse asked me if the room felt stuffy, and requested me to open the windows. A patient, who was sitting a little way off reading her Bible, promptly shut down the first one I opened. I asked her not to do so, and re-opened it. She as promptly slammed it down again. I once more pushed it up, and as she began to try to repeat her performance, I laid my hand upon her arm to prevent her. Without a word she spat in my face, and boxed my ears! To this day I do not know how I restrained the natural impulse to return the compliment. Fortunately the charge nurse had seen the whole occurrence, and at once interfered. She spoke soothingly to the patient, and took her away to another part of the ward. She afterwards told me that the patient was an epileptic, and very passionate, not having the least control over her temper. She also told me to remember that most epileptics were the same. I began to think that the life might be even more exciting than I had at first supposed, and to recognize that more self-control might be needed than I felt I possessed. There was yet another lesson waiting for me. When the matron entered the ward, this patient at once complained that I had been knocking her about, and abusing her. I was struck dumb, but the charge nurse came to my rescue, giving the actual facts of the case. Some time afterwards, when I accompanied this patient to the visiting-room, I had the pleasure of hearing myself named as the nurse who had been ill-treating her. At dinner, when the patients were counted, one was missing. This was a woman who was thought to be recovering, and could be trusted somewhat—she used to assist in cleaning the nurses' rooms, and was made rather a pet of by them. A thorough search was made, but no trace of her could be found. In the evening when one of the nurses went to her room, which was situated in the main corridor, she found that some of her clothes were missing, and presently discovered the patient's garments under her mattress. The method of escape was then evident. When the patient was brought back next day we had a full account of it. She and the nurse were much alike in height and figure. She had put on the nurse's clothes, and, watching her opportunity, had walked out by the unlocked door, and boldly passed the hall porter unnoticed. That evening escapes formed the leading topic of conversation among the nurses, and some of the older ones had very curious tales to relate.

### THE NORTH WALES COUNTIES ASYLUM.

The situation of affairs in this asylum with regard to accommodation for lunatics is very interesting. For years past it has been overcrowded, and, though a branch establishment for 80 females has been opened, there are still 70 patients boarded out at other asylums. Increased accommodation is much needed, but owing to the conflict of authorities there is at present a deadlock. The asylum is provided under an union of five counties, and a body called subscribers. Four of the counties, Denbigh, Flint, Anglesey, and Merioneth, are, along with the subscribers, agreed as to the enlarging of the present buildings. The county of Carnarvon, however, refuses to agree to this, wishing to have another asylum in that county. The present asylum at Denbigh cannot be enlarged without the consent of all parties to the union. Hence the difficulty. The Commissioners in Lunacy and the Home Secretary have been appealed to, and it will be interesting to watch what will come of their action. The committee have acted with coolness and sense under these very trying circumstances.

### SICK NURSING OF THE INSANE.

### Enteric Fever.

The nursing of disease in the insane frequently differs little, if at all, from the same work among those not mentally afflicted. Unfortunately, we meet with many cases of which this cannot be said, and the difficulties with which the nurse has to contend are often very great. If we except surgical cases, the difference between looking after the sane and insane is perhaps as well marked in the nursing of typhoid fever as in any disease. From the very beginning of the attack, the acutely insane, demented, or idiotic, are exposed to special dangers, and the efforts of their attendants peculiarly hampered. Unable to appreciate their condition, or too stupid to make any complaint, it is only when some change in their appearance is noticed that any disease is suspected. Under such circumstances it can be readily understood how enteric fever may have made considerable progress before its discovery. Even among those with all their reasoning powers, the disease may exist for a long time undiscovered, and the individual may walk about during its progress. How much greater then must be the difficulties in the case of the insane, and especially if diarrhœa be absent. Cases are known in which the collapse following upon perforation of the intestine was the first sign of anything being amiss. Generally the out-of-sorts appearance of the patient or an attack of diarrhœa draws attention to him, and an examination is made. Here we must insist upon the importance of the temperature being taken in all cases of sickness or diarrhœa. When the latter does appear, the necessity for scrupulous cleanliness is imperative, and this care must be redoubled when the disease is recognised as enteric fever. We know that this affection is spread by means of the discharges from the bowel, and it will be seen at once how dangerous a source of infection a lunatic afflicted with this complaint might become, should he be one of those classed as "dirty." It will also be readily admitted that a laborious and distasteful task is laid upon the nurse to whose lot it falls to tend such a case. More especially is the work trying when we remember the dirty habits of the patient and how dangerous it is to use poisonous disinfectants, or even to have any materials of such a character in the ward, some of the inhabitants of which are sure to be either mischievous or suicidal. It is perhaps needless to say that all precautions necessary in the case of the sane are equally called for when the patient is a lunatic.

### The American Woman at Home.

(*Continued from page* xxxvii).

BY OUR OWN CORRESPONDENT.

### III.—HER AMUSEMENTS.

"LA REINE S'AMUSE." In America they say, there is no leisured class. This is true of the men, but for many of the women there is abundance of leisure, and they, as the leisured class always will be, are the great amusement seekers. But one thing differentiates the female from the male of this genus; she always talks of these amusements as "her work," her "social duties." The social duties of the American woman seem to be largely owed, or at least paid, to her own sex. If her husband wishes to entertain his friends he is very likely to be told to give them dinner at his club. A late dinner would upset the household, disturb the baby—the baby because a rare is a very dominating factor where he exists—more important than all, it would inconvenience her. When it is a question of a ladies' lunch or tea, things are different; the entertainment is, from the feminine point of view, worth giving. For men—the brutes!—care only for what they eat and drink, and good food and liquor, perhaps better cooking than at home, can he get at an hotel; while women appreciate flowers and fine linen and dainty silver and china. In these things the American housewife prides herself, and with her store of these no club or hotel can compare. The luxury and elaborateness of American table decoration is hardly guessed at on this side. I do not mean that you never meet anything comparable with it, but you certainly do not see it carried to the same extent among people of corresponding position.

American table silver contains many pieces unfamiliar to us—special oyster forks, orange spoons, and cups in which to serve the fruit, a curious sort of pronged spoon, a cross between spoon and fork, dedicated to ice-cream, &c. Every self-respecting woman has her collection of souvenir spoons, brought by herself and her friends from every part of the world. These are always of exquisite workmanship, and are often gilded, enamelled, and even jewelled. The same is true of all the serving spoons and forks, and in a jewellery store in a moderate-sized town you will find a stock of such fancy articles—in good demand too—as it would take our best London shops to rival. Parenthetically, it may be remarked that the care of this will all fall upon a housemaid, who, one would think, had enough to do in polishing hard-wood floors all over the house, and keeping dainty furniture, besides her ordinary routine of sweeping and dusting. A similar fanciful taste prevails in linen. It is a popular fashion, though condemned by some of the most fastidious, to put no cloth on the highly-polished table, but an exquisitely embroidered centre-piece, and d'oyleys to match for each guest. The cost of these may reach any point your purse permits, and they are frequently real works of art. Drawn threadwork of cobweb fineness, imported from Cuba or Mexico by the rich, done at home with much sacrifice of eyesight by those less moneyed, is also popular. A lady of my acquaintance has just completed, as the result of five years' leisure-work, a table cloth and eighteen table napkins with borders of drawn threads caught and twisted into every conceivable pattern, upon which triumph her husband comments thus: "I can't see why women buy good linen just to pull it to pieces again." Doubtless the labour has a market value, though disproportionate to the work. I was asked 30 dollars (£7 10s.) for a moderate sized piece three-quarters of a yard square with only the corners worked. The value of embroidered linen also rises to a high amount. One set of cloth and napkins which I saw embroidered in roses caught with ribbons was valued at £200. Coloured cloths of all kinds are quite *comme il faut* at luncheons, though to an English taste they are less attractive than plain white damask.

Flowers are, of course, another expensive item. Floral decorations at the rate of £1 for each guest are by no means extravagant, as things go there. In fact the aim of the American luncheon party is to show to those who can appreciate—*id est*, other women—the wealth, and, secondarily, the taste of the hostess. The food is more dainty than solid, and it is a popular fancy to keep up some one idea throughout. Everything possible, for example, is served in baskets—baskets made not only of willow but of pastry, of candy, of the paste from which cheese-straws are often made, of anything that can be made accessory to the course in progress. Or at an Easter lunch everything will either, in shape or substance, suggest eggs. It is on the ingenuity with which these fancies are carried out that the success of the entertainment depends, and as each new surprise comes on exclamations of admiration gratify the hostess's vanity. Sometimes, in default of surprises, conversation may languish, and then various aids may be provided. A conundrum, or quotation salad may be introduced. On lettuce leaves made of green crinkled paper, and artistically arranged in a china bowl, the riddles or quotations are written. The hostess has in a book the key to answers or authors' names, and the one who guesses most receives a prize, perhaps the bowl in which the salad was brought in, or some pretty, and by no means costless trifle. The souvenir is still, though some people condemn it as foolish and ostentatious, a prominent feature of American entertainments. Often the guests are expected to carry away the little "individual" dishes in which bon-bons are served. These may be little baskets, pretty enough, but inexpensive; but they may be of china or silver if the hostess can afford to make handsome presents. No card party would be complete without its prizes, prizes for the most successful, "booby" prizes for those at the other end. The commercial element cannot be eliminated in America even from hospitality.

The luncheon is a more or less intimate affair—limited in the number of guests; the great reception, what we should frankly call a "crust," is the ladies' tea. Her notion of an afternoon tea is something friendly and informal, cosy chats with intimates, and cups carried by the guests themselves to any corner they choose, but such is the American tea. You go through your visiting list and invite two or three hundred people. These, having shaken hands with their hostess, are passed on to the dining-room. Here, however, no crowd is permitted. The available seats are filled, those who come after must wait until those who have gained admittance are fed and depart refreshed. Solemn coloured waiters administer salad, bread and butter, coffee or chocolate, and cake (tea is often conspicuously absent from a "tea"). You aren't allowed to help yourself nor encouraged to stand about, cup or plate in hand, talking with your friends; and, indeed, you cannot but be conscious of the hungry crowd waiting outside until your wants are supplied. So you pass on to another apartment where ice-cream is served, and, perchance, to another where you get lemonade. But all is formal, and the absence of men—the good, clumsy creatures who can never help putting in a touch of *bonhomie*—makes the entertainment serious to the last degree, in spite of the daintiest of bonnets and gayest of gowns.

Entertainments in America mean entertainments for women. Even in the theatre this is so, and the *matinée* has an importance we cannot realise, for madame goes to that, and then decides if the play is one to which she can conscientiously take her husband. Perhaps there are plays he goes to without consulting her, but when she takes the initiative he never refuses to follow.

### Orphans in Cages.

The pamphlets and appeals issued broadcast by the Church Extension Association make *Our Work*, as the Kilburn Sisters call their own monthly magazine, a very familiar sight to most of our readers. It is full of good schemes and useful plans, and arouses as much interest as could be wished even by the kindly band of workers in the great pile of buildings at Kilburn. The invariable courtesy of these ladies render a visit to their orphanage a memorable event to many strangers. Whether this system of massing together many hundreds of children and making them into details of the vast machine of which each fragment is dependent on external support, and is, therefore, unable and unfit to stand alone, is altogether wise, is perhaps, open to discussion. Untrained in habits of independence and self-reliance, orphan girls must surely find an undue amount of temptation awaiting them in the world without these walls. There is, however, a constant danger within the home to which immediate attention is now called for. Unless it is abolished we shall some day hear of a terrible catastrophe unparalleled in history. The children when in bed are locked up in wire cages. The accompanying illustration will make our meaning plain. The bar on the outside secures all the doors of the whole row of cages. Hence no child can liberate itself until the lever is raised which frees them all. In a dormitory containing, say, 50 or 60 beds, there are at least four levers to be worked before all the orphans' doors are unfastened. A "sister" sleeps in a room opening into the ward; but what can a single terrified woman do in a case of fire or other catastrophe, which under such conditions would well nigh paralyse a strong man? If those in authority at the Kilburn Orphanage cannot get sufficient "workers" to ensure more personal supervision for the orphans, it will be well for them to institute a less dangerous expedient for keeping children in order. "It reminds me of a penal settlement," said a gentleman going round the home lately; and truly there must be deterioration instead of progress under discipline enforced by this kind of compulsory detention. Cubicles? Yes, by all means, cubicles, but let them have open doors. When children learn they must keep in their own divisions as an act of voluntary obedience, their minds and their morals will reach a higher plane than is possible during their present nightly incarceration within iron walls and spiked roofs. Even little invalids are enclosed after the same fashion. A kind of small panel in the side of the cage on a level with the pillow being so arranged as to open from the outside. Thus enabling the attendant to serve the patient with nourishment, etc., without opening the door of the cage. There is, perhaps, neither cruelty nor discomfort in the present scheme, but those who believe it is good merit pity rather than blame.

### Nursing in Ireland.

### THE MATER MISERICORDIÆ HOSPITAL, DUBLIN.

The Mater Misericordiæ Hospital is by far the largest general hospital in Dublin. It is situated on the north side of the city on elevated ground, and is a most imposing building, classical in style. It was founded by the Sisters of Mercy in 1852, and was opened eleven years later. The first completed portion of the building contained accommodation for about 40 patients, the east and west wings not being opened until 1872 and 1884 respectively. There are now beds in the hospital for 320 patients, and they are nearly always full.

At present there are isolated wards for fever cases, for whom 50 beds are reserved; but it is intended as soon as possible to build another hospital solely for fevers.

The Mater Misericordiæ Hospital also possesses accommodation for 12 private patients in a house in Eccles Street, annexed by the hospital for this purpose. A sister presides over each ward, and also over each department, such as the kitchen, laundry, &c.

The nurses—or rather probationers—40 in number, are under the supervision of Miss McGivney, the Matron, who was herself trained in the London Hospital. The nursing school has only been established about three years; in fact, the first set of probationers have just completed their course of three years' training. They appear to have enjoyed exceptional advantages, the members of the consulting staff giving them a medical and a surgical lecture each week, and the obstetric physician also undertaking a course of lectures.

The Nurses' Home is in Eccles Street, within easy reach of the hospital, and here the probationers are made as comfortable and happy as possible. They are under the control of the sisters, and under the direct personal and professional superintendence of the matron. There are in the hospital three resident surgeons and one physician, as well as eight resident pupils, each of the latter holding office for six months.

The Pathological Museum is one of the most noteworthy parts of this institution, and invaluable to the students, of whom there is always a large attendance at the important medical school connected with the hospital. Punctually at nine a.m. the hospital is visited by the physicians and surgeonson duty, the students accompanying them in their visits to the wards.

The garden and grounds are very extensive, and form an attractive recreation resort for convalescent patients. Since its first foundation, under the able and kindly superintendence of the Sisters of Mercy, the hospital has been doing an infinite amount of good in the relief of suffering, and the recent establishment of a training school for nurses cannot fail to greatly increase its value

### Wants and Workers.

Nurse Emily Halford will be glad if any Hospital reader can give her the present address of her old friend, Nurse P. Clayton.

Can anyone tell me of a home where an idiot girl of six could be received for about three shillings a-week? The parents are too poor to pay more. The child's health is good, but she is very, very helpless, and can neither stand nor walk. Address "Pity," care of Editor.

For Old Time Nurses, Nursing on the Labrador, Everybody's Opinion, &c., see page lxi, et seq.

### Old Time Nurses.

(*Continued from page* ccxv., *Vol.* xvi.)

### IV.—LONG SERVICE.

It is a common complaint that competition in the nursing world becomes every year more keen. Those who make this complaint forget, however, that side by side with the rapid increase in the number of nurses there is a marked and steady increase in the number of those who enlist their services. To "have in" a nurse was considered only a few years ago the luxury of the very wealthy. In the present day the nurse is an ordinary presence in any middle-class household. The kitchen or house maid who formerly numbered among her duties a casual superintendence of the paralysed or invalided member of the family has been very generally displaced by the regular attendant; and though in such households "nurse stories" are apt to form one of the staples of conversation, the comfort of leaning upon skilled assistance is too real to be relinquished. The demand even for the untrained or old-time nurse if not altogether equal to the supply is not very greatly behind it, and is likely to increase rather than diminish.

The difficulty experienced by many capable women in getting work rests upon many causes independent of the laws of supply and demand; and that this is so, appears from the corresponding difficulty of securing the right person as attendant, commonly experienced by those responsible for the care of an invalid.

There is little doubt that long terms of service, however grateful to the patient and creditable to the nurse, constitute a serious impediment to the after advancement of the nurse. It is not only that the working connection is completely dropped in the course of a service of some years, but the nurse is apt to lose some of that adaptability to circumstances and readiness to undertake the unfamiliar which are necessary parts of her stock in trade. When the long familiar routine is abruptly broken short she finds herself helpless in a world from which she has long been absent; she is filled with distrust of her capabilities under new circumstances; she is easily discouraged and entirely vague as to the best means of procuring fresh work, and it commonly ends in a long period of heart-sick expectancy, during which the savings vanish and destitution approaches very near. These facts should he fully realised by all those who cling to the ministrations of some familiar attendant, knowing all their ways, and, in the common phrase, "an invaluable treasure." Too often cases occur where the nurse surrenders the best years of her life to self-sacrificing labours, to find herself cut adrift in the end with nursing abilities crippled by long disuse, and with savings entirely inadequate to form a provision. The writer has in mind a pathetic case of this description. Nurse L. is one of the timid, gentle "old time" nurses fast disappearing from the profession. The slow, distinct articulation tells of many years association with the aged. The nervous manner, the diffident self-effacement, and the simplicity revealing every passing emotion in the face, and knowing no disguise, point to a life spent entirely out of the world. It is easy to read in the lines of the little apple-cheeked face, and in the steady brown eyes the story of peaceful, guarded years, spent in the performance of always the same duties, lifted out of the commonplace by a rare touch of self-devotion and tender gratitude. Early in her nursing career Nurse L. had taken service with an invalid lady living with a friend. For some thirty-five years she remained in the same service. In course of time, partly from old age, the friend also became an invalid, and the nurse waited upon both with a deep attachment, growing year by year more pathetic as it became evident that they were passing slowly away from her care. Neither lady had more than a small annuity to depend upon, and the fate of their faithful attendant became a matter of the deepest anxiety to them. At the very first intimation in the newspapers of the formation of a pension fund for nurses they set about procuring information, and owing to their activity Nurse L.'s application was among the first received. The whole of her savings were promptly invested in the fund, and to this was added what could be spared from their scanty income. Two or three years later the old ladies died, one soon after the other, leaving their old friend and nurse the scanty stock of furniture and few personal possessions which made up their effects. Her position, sad enough in the entire absence of friends or relatives, and unfitness for undertaking other work, has still in it an element of hope. With a well-furnished room and a habit of ceaseless industry, she is able to get through the next two or three years partly by doing needlework and partly by falling back in slack times on possessions which, though superfluous, are most reluctantly parted with. She will then enter upon a period of complete independence and freedom from anxiety, the fruit of all the past years of happy industry. The ceaseless struggle to procure daily necessaries can be cheerfully borne with the goal of security in sight; but what if the struggle must continue with failing powers, and the workhouse rising ever more distinct in the near future?

Nurse R. is in unhappy contrast with the nurse just quoted. The daughter of a clergyman, she has pursued the calling of a nurse for nearly half a century. Now at an advanced age she is still fighting off infirmity, desperately struggling to get work, sorely overtaxed by it when obtained, miserably pinched in the long intervals between each situation—intervals which have long ago consumed the savings of more prosperous times—worse than all, gnawed by incessant anxiety. She illustrates only too forcibly the responsibility which lies on the employer, in these cases of chronic patients (which have mostly fallen to her share) with regard to the future of the nurse. It is a responsibility seldom recognised in its fullest extent. "She has been with us such for such a number of years," says the family with a somewhat justifiable pride. "She is quite devoted to poor A., and while he lives we should never part with her." Then "poor A." dies, and the nurse who for £20 or £30 a year has devoted herself, body and soul, during the best part of her life to him, receives a suit of mourning, and perhaps a present of £10, or it may be only her patient's watch and photograph. The family mean to be very kind. They tell her they will never lose sight of her, and in any great season of distress she may probably count upon them for a postal order. The evil is that they do not in the least realise either their own obligation or the difficulties of her position, and how every additional year of service with them, so far from being the benefit they had imagined, was placing her at a more serious disadvantage towards her profession.

The case already cited of Nurse L. proves that the solution of the difficulty does not, as many people think, lie in the wealthiness of the patient. A legacy to the nurse, sufficient to secure her future from want is not a very frequent occurrence. It is open, moreover, to the double objection of awakening jealousy on the part of the relatives, and of giving a loophole to that subtle suspicion on the part of the patient, common in lingering illness, that his death will not be altogether unwelcome. Cases can be cited, of course, where the services of an old attendant are wisely and justly rewarded by a pension. But even among rich people a donation is the more common acknowledgment, and it has been shown that no class of persons have less skill in placing their money to good advantage than nurses. For an ordinary middle-class family with limited means, neither pension nor large donation is possible, however much it may honestly be desired.

What is fully within the reach of all is to ensure, by a system of co-operation with the nurse herself, that she shall not in later life fall into want. The long term of service does at least enable her to lay something by, although this something is apt to dissipate only too speedily on her departure. As her term of service lengthens and it becomes evident that no one else can so fitly supply her place in the household, let it be an indispensable condition of her service that some part of her salary be devoted to securing a pension in the future, and let a certain proportion of the premium, according to the means of the family, be contributed yearly by those to whom she is devoting her care. If this could become the regular practice, every year of faithful attendance would advance the nurse's prospects of future independence, and long service, instead of leading to inevitable future misery, would be as greatly to the advantage of the nurse as of the patient.

### Nursing on the Labrador.

(Communicated.)

### MISSION TO DEEP SEA FISHERS.

Indian Harbour.

Our life here can never be monotonous. One night lately we had a gale of wind lasting until morning, causing our little hospital to rock so much that we lay awake wondering what would happen to it and to us. Next day the first operation was performed, the removal of a large tumour. The night watches have to be divided between nurse and doctor, and when there are urgent cases everyone has plenty to do.

Great help has been given to the mission by ladies at Montreal, who take a deep interest in the work, and cases of clothing, blankets, sheets, &c., sent by them have been unspeakably welcome, for the things were sorely needed.

Another girl has come to assist in the rough work; her father was English, her mother a native of Labrador. She is very dark complexioned, with jet black hair, and of her English descent she is excessively proud. Once a year the relations she has never seen write to her.

Perhaps readers of The Hospital will like to know a little of our actual work, for this comprises a curious variety of duties. For example, our "helps" are most helpless young women. In spite of repeated lessons in "setting the table," our brightest girl still puts knives to the left and forks to the right hand; and if we waited for breakfast until either of them prepared it we should have a long fast. So that getting breakfast for the whole party (not only the patients) is left necessarily to nurse; then she makes beds, and in the meantime the girls peel potatoes and wash up.

The absence of fresh meat (we ate it last at Swansea) and green vegetables has a confining influence on our cooking. A gift of turnip tops made quite a festival one day. One of the girl "helps" proved a good bread maker, and for this we were daily grateful, but she did not stay long, having an ambition to go to Newfoundland for the winter.

Remembering the amount of work an ordinary English wardmaid accomplishes as a matter of course, the slowness of the women here is almost insupportable, but yet assistance in the rough work is too essential a comfort for us to do otherwise than accept gratefully such "helps" as we obtain.

On Sunday we have service at 11, nurse playing the hymns on her "baby organ," of which a native remarked admiringly that "he'd never seen or dreamt of the like of it." After service comes the dinner (prepared and served under nurse's supervision). All the afternoon patients stream in, and by the time they have been seen there is barely time for tea before evening service. Some of the patients are bad enough to be taken in at once, and need nurse's immediate services. The washing of sick folks is not an enviable duty when they don't change their stockings during the whole season!

The "Windsor Lake" called at Indian Harbour on her way south, bringing one of our doctors who had with him a man suffering from acute eczema. His hands were hard and dry, with deep cracks on the fingers almost down to the bone. He had been washing salt fish in salt water, and his skipper offered strong opposition to his leaving his work to come to hospital! He must have suffered terribly. He said he had tried every remedy he could hear of, including oatmeal and vinegar, and finally tar, which he described as having "a'most burnt the flesh off his bones!"

On one very cold day the doctor was fetched to see a young man at Horse Harbour. He found him in a very draughty "bunk house," with no one to look after him except at "scattered times," as the poor fellow said, when the men came in from fishing, and he was suffering from bronchitis and double pneumonia; so he was wrapped up and brought away to our tiny hospital, where he seemed thankful to find himself in a comfortable bed, getting nourishment every two, and medicine and brandy every four hours. His temperature was 104 degrees. The first patient we ever had here helped to convey this one, and he took an opportunity to say, in a quivering voice, "How can I ever love and thank you for all that was done for me in this place!"

### Should Fever Nurses be Trained?

Last Saturday the Nursing Staff Committee laid a scheme before the Metropolitan Asylums Board which called forth discussion and opposition, its consideration being eventually postponed for six months. Its purport was that probationers might be received in the Board's hospitals for two years' systematic training in fever nursing, at salaries of £20 for the first and £24 for the second twelve months. It was suggested that this would obviate the disadvantages resulting from the present constant changes in these institutions. Those who opposed the plan seemed to think London would be overcrowded by "a new class of woman" bearing the stamp of the Metropolitan Asylums Board; but the "stamp" might possibly proclaim merely a valuable distinction between a few weeks' "experience" and those well qualified in one branch of nursing. The proposal to admit women from 21 years of age would, whilst creating a supply of efficient nurses for fever cases, also leave many free, on the completion of their two years' agreement with the Board, to continue in a general hospital or infirmary the study of the art of nursing, for only in these institutions can be secured complete training in all branches. Many girls would be glad to make the practical beginning offered by work in a fever hospital at a salary, whilst waiting till their age permitted of their taking up general training. Perhaps, at the expiration of the six months' postponement, further consideration of the scheme will show that adequate training in any one branch of nursing is not to be confounded with the three or six months' course; in the latter all varieties of nursing are nominally learnt, and such courses do far more to flood the country with incompetent people than would be likely to result from any well-organised scheme for giving full training in fever nursing to suitable women.

### Workhouse Infirmary Nursing Association.

This hard-working association has cause to congratulate itself on the pleasant signs of progress recently evinced by sundry Boards of Guardians. For instance, at Plymouth it has been decided to engage a superintendent of nurses, at £50 per annum, and three trained nurses, from the Workhouse Infirmary Nursing Association. Barton Regis Guardians have for many years had nurses from the association, but the staff has been hitherto inadequate in numbers, and we are glad to learn that it is to be increased to seven, including the superintendent-nurse. After many years of earnest work at Hastings, a lady Guardian has been instrumental in persuading the Guardians to have a trained head nurse, a trained assistant, and a probationer. This is a very fair staff for 56 beds, 45 being the average number occupied. Much remains to be done, but each sign of improvement is hopefully welcomed; and we strongly advise earnest girls who want to be trained and superior nurses, willing to devote themselves to infirmary work, to go and see the hon. secretary of the Workhouse Infirmary Nursing Association, 6, Adam Street, Strand.

### Appointments.

Camberwell District Nursing Association.—Miss E. Chadwick has been appointed Superintendent of this home, which is affiliated with the Queen's Jubilee Institute. She was trained at the Royal Southern Hospital, Liverpool, where she remained for three years. Miss Chadwick was trained in district nursing at the Central Home, Bloomsbury, and acted as Superintendent-Nurse at Darwen, Lancashire, where her admirable work was highly appreciated. We wish her every success in the new post, for which her experience has especially fitted her.

### Minor Appointment.

Boscombe Cottage Hospital and Provident Dispensary.—Miss Ethel Lawrence, who trained at the Sussex County Hospital, has been appointed Nurse-Matron of the Boscombe Cottage Hospital. Miss Lawrence has also worked at the Lewes Infirmary and Victoria Hospital, and we congratulate her on her appointment.

### Everybody's Opinion.

[Correspondence on all subjects is invited, but we cannot in any way be responsible for the opinions expressed by our correspondents. No communications can be entertained if the name and address of the correspondent is not given, or unless one side of the paper only be written on.]

### THE RIVIERA.

"A Correspondent" writes: Can any reader of The Hospital tell me of a home in the Riviera for consumptive patients of small means? I shall be very glad of information for a man who has been recommended to go to the South of France for the winter. He could not afford the usual terms charged. He is a most respectable man, and perhaps there may be some moderate establishment known to and recommended by some of your readers where he could afford to go.

### POOR LAW INFIRMARY MATRONS AND MEDICAL SUPERINTENDENTS.

"An esteemed and experienced correspondent" writes: I was very interested to read your paragraph about the Lewisham Infirmary. It opens up a most important point which I hope The Hospital will deal with, and that is the relations between the Medical Superintendent and Matron in a Poor Law Infirmary. Their duties are of a most conflicting kind, and the difficulty, as things now stand, is to avoid friction. The rules laid down by the Local Government Board for the Matron are exactly the same as those which were in force years ago when she was expected to be a housekeeper and nothing else. Modern requirements expect her to be a trained nurse as well, but give her no supervision or control over her department except what the Medical superintendent chooses to permit. As Miss Twining has pithily expressed it "the position of a matron in a Poor Law Infirmary is exactly what the medical superintendent chooses to make it." If you get an overbearing, inexperienced man to deal with, the result is not difficult to foresee. So long as things remain as they are so long will these scandals arise. You cannot expect a trained and educated lady to have no opinions, to be a cipher in her own department, and carry out any orders, right or wrong, given her by a very young man, often inexperienced and with other drawbacks in addition. I have heard so often of troubles arising through the absurd system at present in force, and I am glad Miss Pattison has the pluck to come forward as perhaps it will lead to the necessary reforms being made. There is no doubt the matron should be responsible to the committee for the control of her own department, and that her duties should no more clash with the medical superintendent than they do in a general hospital. I do hope the matter will be warmly taken up; there is a very strong feeling about it, but the movement must come from outside, as for obvious reasons officials cannot put themselves forward prominently.

"A Country Matron" writes: Until more is known of the breach that has taken place at Lewisham Infirmary it would be premature to judge; but the conclusion of the matter will be most anxiously watched for by all matrons holding like positions. Should the Local Government Board not realise the gravity of the issues, it may mean debarring any intelligent lady from seeking or holding a post which places her authority over the women of the building under an officer who may or may not have the capacity and character to rule the opposite sex I cannot altogether agree with last week's "Nursing Mirror." The two officials have distinctly different horses to ride, and I am glad for the brave and independent spirit of the Lewisham matron in aiming a blow at one of the greatest abuses in the nursing world. Although I am only matron of a country hospital—just over 100 beds—yet I am thankful to possess the following amongst my rules: "The matron shall be at the head of and accountable for the whole female department, and shall report cases of misbehaviour or neglect to the Hospital Committee." So far as the medical treatment of the cases is concerned the doctor must regulate the nurses' duties, but the matter ends there. The matron should be also the head nurse, and any complaint of inefficiency lies with the capacity of the matron to manage her nursing and those under her. In disciplinary matters as regarding the women, the medical superintendent should give way to the matron, as she on her part must, on a much larger scale, own her dependence on the medical superintendent's larger sphere of action.

### For Reading to the Sick

### HEAVEN.

**Motto.**

However forgotten, heaven still is thy home.—*Boethius.*

Those who are bound for heaven must be willing to swim against the stream.—*Matthew Henry.*

In My Father's house are many mansions; if it were not so I would have told you; I go to prepare a place for you . . . . and I will come again and receive you unto Myself.

**Verses.**

Come to the Land of Peace!
Come where the tempest hath no longer sway—
The shadow passes from the soul away—
The sounds of weeping cease!
Fear hath no dwelling there.
Come to the mingling of repose and love,
Breathed by the silent spirit of the dove
Through the celestial air! . . . .
In thy divine abode,
Change finds no pathway, memory no dark place!
And oh! bright victory—Death by Love no place!
Come, spirit to thy God! —*F. Hemans.*

Alone? The God we trust is on that shore,—
The Faithful One Whom we have trusted more
In trials and in woes, than we have trusted those
On whom we leaned most in our earthly strife.—*Faber.*

**Reading.**

A merchant maketh far voyages and great journeys, and ventureth body and goods, and nothing is too hard and sour for him, only for worldly gain and lucre. And yet his hope is uncertain whether his chance shall be good or evil. And though he happeneth never so well, yet he bringeth home nothing but frail and transitory goods which shall have an end.

Now, all *we* have a long voyage to make also, even from earth to heaven. And should not we be as well content, as prompt, and glad and willing to suffer all manner of perils and dangers that may happen by the way, seeing that we have an infallible and sure hope of eternal and everlasting riches for Jesus Christ's sake?

A wayfaring man that goeth from home, although he passeth many pleasant houses and goodly meadows, yet minding altogether his home, all such things do nothing tempt nor grieve him; even so, whensoever we have not all our pleasure and delight here, let us establish our comfort, and delight ourselves with our country and habitation in heaven (2 Cor. v.; Phil. iii.).—*Bishop Coverdale*, 1550.

Fainting soul, failing for fear at the thought of death, haunted by its awful shadow . . . sorrowing heart, is there no word of comfort for thee in the Gospel of love? Yes, thy blessed Master . . . has left a message for thee, "Let not your heart be troubled . . . I go to prepare a place for you." . . . Doubt not O watching, waiting soul that this promise is for thee. . . . It may be that the last journey so dreaded in life will be the sweetest thou didst ever take, for it will be with Him; it may be that it will be dark, but what matters the darkness if thou canst feel His hand; it may be hard and toilsome, but what is that . . . if only He has come for thee to be thy guide even through death.—*M. E. Townsend.*

### Notes and Queries.

**Queries.**

(46) *Patent.*—Can you tell me where to get an appliance patented?—*Sister Ethel.*

(47) *Registers.*—Can you inform me where medical men were registered in 1700; is there any list of them to be seen anywhere?—*A. C. C.*

**Answers.**

(46) *Patent* (*Sister Ethel*).—You had better write to the Institute of Patent Agents, 19, Southampton Buildings, London, W.C.

(47) *Registers* (*A. C. C.*).—By the provisions of the Apothecaries' Act, passed in 1815 a fine was imposed on any who acted as apothecaries without due license from the Society of Apothecaries (*i.e.*), the L.S.A., but all who were in practice as apothecaries before that date were exempted from its provisions. In 1858 the Medical Act was passed (21 and 22 Vict. c. 90), which arranged for registration of all who had certain qualifications as well as those still remaining who were in practice prior to 1815. Since 1858 there has been a legal distinction between qualified and unqualified practice, but before that there was no register, except of course the lists of the various qualifying bodies, which appear to have had no legal force except in the case of the apothecaries.

## Figures and Tables

**Figure f1:**